# Spred2 Deficiency Exacerbates D-Galactosamine/Lipopolysaccharide -induced Acute Liver Injury in Mice via Increased Production of TNFα

**DOI:** 10.1038/s41598-017-18380-0

**Published:** 2018-01-09

**Authors:** Xu Yang, Masayoshi Fujisawa, Teizo Yoshimura, Toshiaki Ohara, Miwa Sato, Megumi Mino, Thar Htet San, Tong Gao, Steven L. Kunkel, Akihiro Matsukawa

**Affiliations:** 1 Department of Pathology and Experimental Medicine, Graduate School of Medicine, Dentistry and Pharmaceutical Sciences, Okayama University, Okayama, Japan; 2 Department of Pathology, University of Michigan Medical School, Ann Arbor, Michigan USA

## Abstract

Acute liver injury (ALI) is characterized by hepatocyte damage and inflammation. In the present study, we examined whether the absence of Sprouty-related EVH1-domain-containing protein 2 (Spred2), a negative regulator of the Ras/Raf/ERK/MAPK pathway, influences ALI induced by D-galactosamine (D-GalN) and lipopolysaccharide (LPS). Compared to wild-type mice, Spred2^−/−^ mice developed exacerbated liver injury represented by enhanced hepatocyte damage and inflammation. Enhanced ERK activation was observed in Spred2^−/−^-livers, and the MEK/ERK inhibitor U0126 ameliorated ALI. Hepatic tumour necrosis factor α (TNFα) and interleukin (IL)-1β levels were increased in Spred-2^−/−^-livers, and the neutralization of TNFα dramatically ameliorated ALI, which was associated with decreased levels of endogenous TNFα and IL-1β. When mice were challenged with D-GalN and TNFα, much severer ALI was observed in Spred2^−/−^ mice with significant increases in endogenous TNFα and IL-1β in the livers. Immunohistochemically, Kupffer cells were found to produce TNFα, and isolated Kupffer cells from Spred2^−/−^ mice produced significantly higher levels of TNFα than those from wild-type mice after LPS stimulation, which was significantly decreased by U0126. These results suggest that Spred2 negatively regulates D-GalN/LPS-induced ALI under the control of TNFα in Kupffer cells. Spred2 may present a therapeutic target for the treatment of ALI.

## Introduction

Acute liver failure (ALF) is a life-threatening illness and its management remains a significant challenge. ALF is the most severe form of acute liver injury (ALI) that typically manifests as hepatic dysfunction, coagulopathy, and encephalopathy^1^. ALI can be induced by various factors including viruses, drugs, and toxins^1,2^. ALF is associated with sepsis^1^, and there is a high incidence of bacterial infection in the early phase of ALF^3^ resulting from impaired phagocytic function and reduced complement levels^4^. Mortality in patients with ALF increases as the magnitude of systemic inflammatory response syndrome caused by sepsis increases^5^. Sepsis is frequently caused by gram-negative bacteria, in which lipopolysaccharide (LPS), a toxin in the outer cell membrane of gram-negative bacteria, induces a wide variety of inflammatory mediators including cytokines. These results indicate that LPS plays a role in the progression of ALF under specific conditions.

D-Galactosamine (D-GalN)/LPS-induced ALI in mice is a well-established experimental hepatitis model^6^. D-GalN is an amino sugar that blocks RNA synthesis and greatly increases the sensitivity of LPS-induced hepatotoxicity^7^. Hepatotoxicity largely depends on inflammatory cytokines, particularly tumour necrosis factor α (TNFα)^8^ produced after LPS stimulation via Toll-like receptor 4 (TLR4)^9^. TNFα activates not only nuclear factor (NF)-κB but also the mitogen-activated protein kinase (MAPK) pathway^10^. The MAPK pathway is composed of extracellular signal-regulated kinase (ERK)-1/2, p38, and c-jun N-terminal kinase (JNK)-1/2^11,12^. Recent studies showed that MAPKs are involved in the inflammatory response during ALI. A JNK specific inhibitor, SP600125, and inactivation of MAPKAP kinase 2, a target of the p38-MAPK, protected mice from D-GalN/LPS-induced ALI^13,14^. Robust ERK activation was observed in D-GalN/LPS-induced ALI^15^. Considering the involvement of ERK-MAPK in liver pathology, it is reasonable to speculate that ERK-MAPK plays a role in ALI. Dysregulation of ERK-MAPK may exacerbate D-GalN/LPS-induced ALI.

The signalling pathway is counterbalanced by endogenous mechanism(s). An imbalance in the cytokine response may result in irreversible liver injury. Sprouty-related EVH1-domain-containing proteins (Spreds) are a family of proteins that inhibit Ras-dependent ERK signaling^16^. As the ERK-MAPK pathway is activated in D-GalN/LPS-induced ALI, endogenous Spred proteins may be involved in regulating immune responses. However, the physiological functions of Spred proteins during ALI remain unclear. Spred1 and Spred3 are selectively expressed in the brain and cerebellum, whereas Spred2 is ubiquitously expressed in various tissues, including the liver^17,18^. We recently demonstrated that Spred2 deficiency exacerbated LPS-induced lung inflammation with increased leukocyte infiltration by up-regulating the ERK-MAPK pathway^19^. In this study, we investigated the role of Spred2 during the course of ALI. We demonstrated that Spred2 controls the development of D-GalN/LPS-induced ALI by negatively regulating the ERK-MAPK pathway.

## Results

### Spred2 deficiency exacerbates D-GalN/LPS-induced ALI

**Figure 1:**
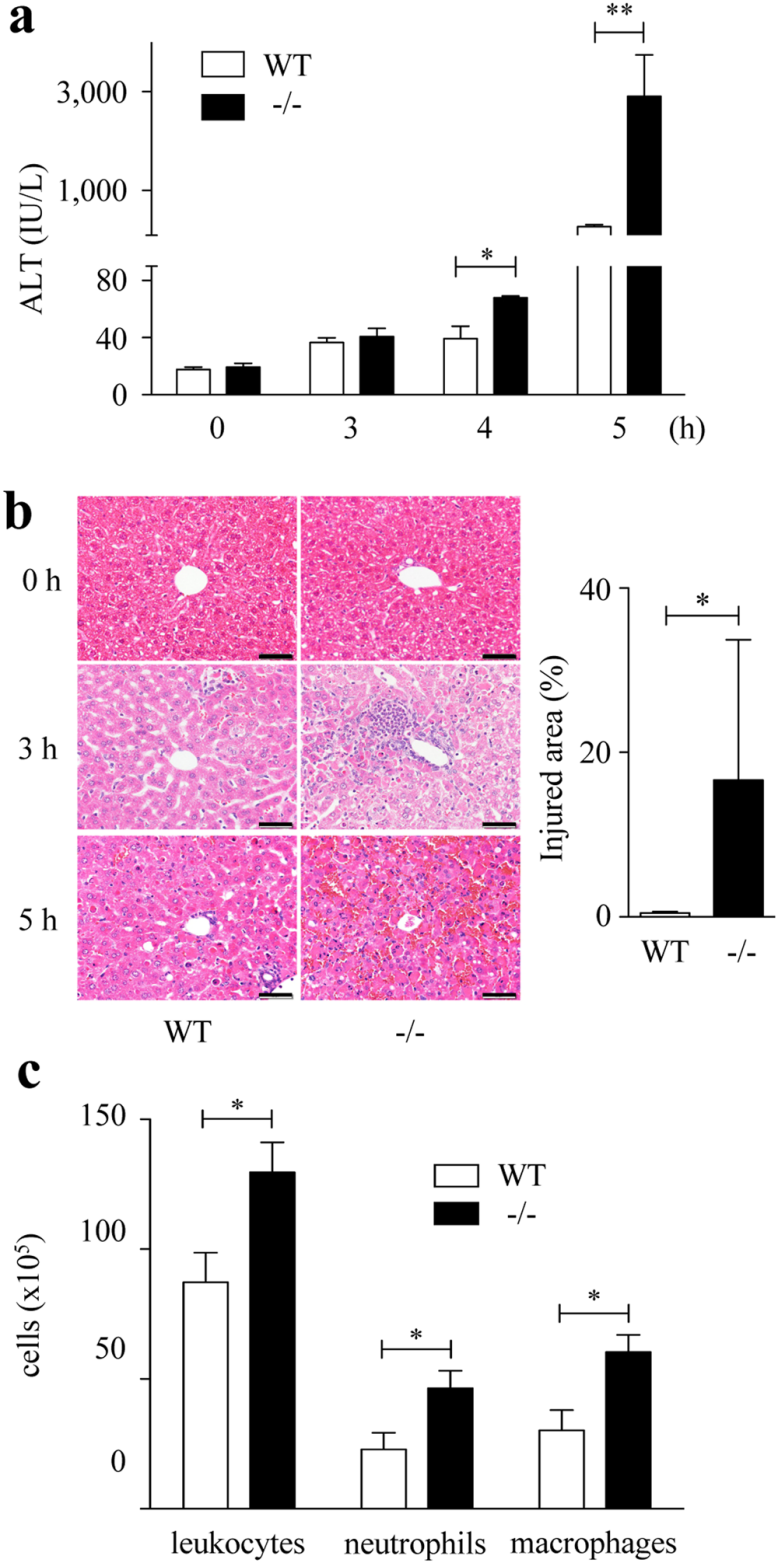
D-GalN/LPS-induced liver injury. WT and Spred2^−/−^ mice were i.p. injected with D-GalN (400 mg/kg)/LPS (20 µg/kg). (**a**) Serum ALT levels were measured after D-GalN/LPS challenge (3–10 mice, each time point). *P < 0.05, **P < 0.01 vs. WT mice. (**b**) Left, representative photographs of liver HE sections at indicated time after D-GalN/LPS challenge are shown. The scale bar indicates 50 µm. Right, injured area with haemorrhage at 6 h after D-GalN/LPS challenge was semi-quantitated (6 mice, each). *P < 0.05 vs. WT-livers. (**c**) Hepatic leukocytes at 3 h after D-GalN/LPS challenge were isolated from WT (6 mice) and Spred2^−/−^-livers (8 mice), and stained with PE-Cy7 anti-mouse CD45 (leukocytes), FITC anti-mouse Ly6G and PerCP/Cy5.5 anti-mouse CD11b (neutrophils), or PE anti-mouse F4/80 and PerCP/Cy5.5 anti-mouse CD11b (macrophages), and analysed by flow cytometry. *P < 0.05 vs. WT mice.

**Figure 2:**
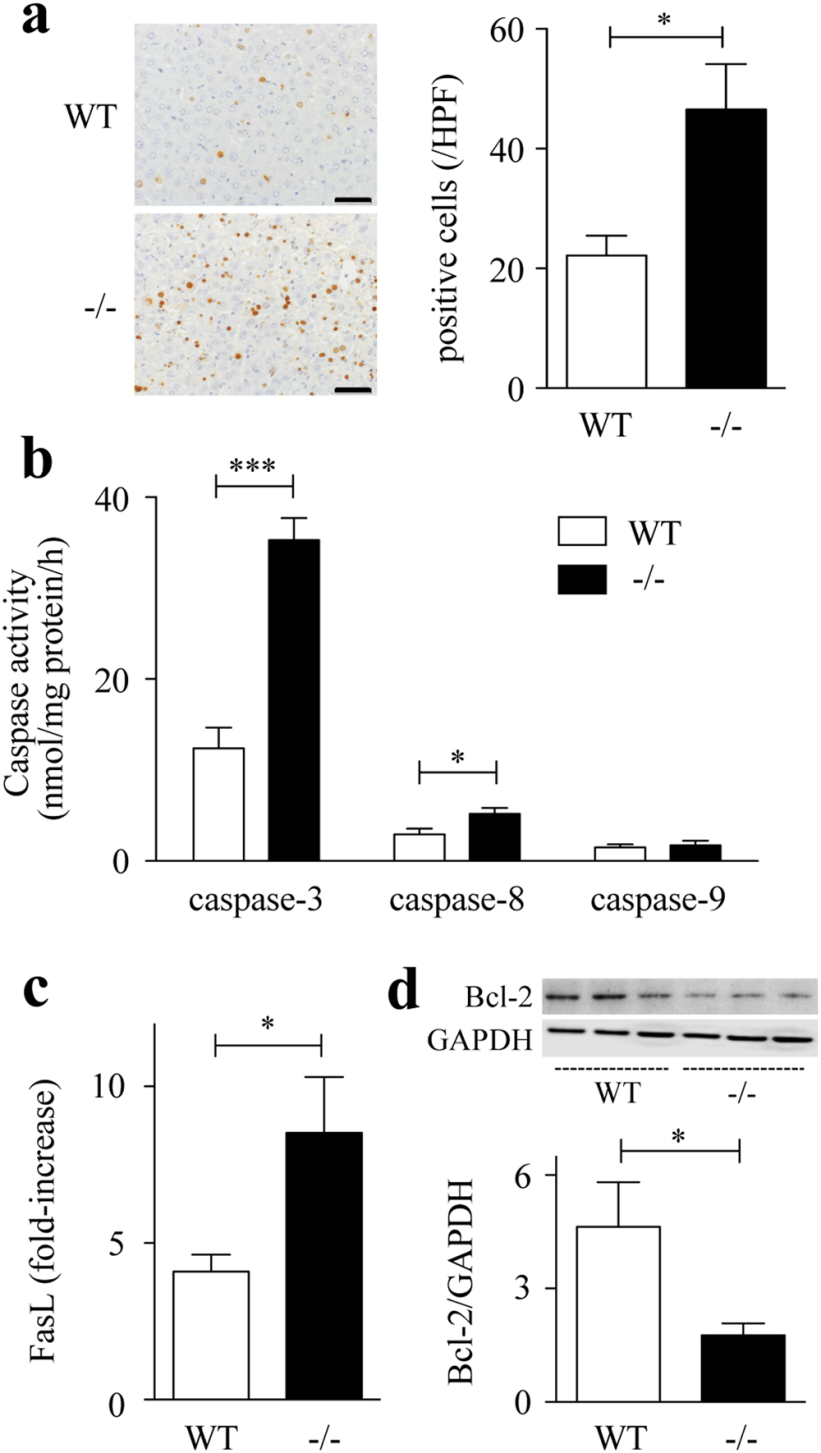
Hepatocyte apoptosis in D-GalN/LPS-induced liver injury. WT and Spred2^−/−^ mice were i.p. injected with D-GalN (400 mg/kg)/LPS (20 µg/kg), and the mice were sacrificed at 5 h after injection. (**a**) Left, representative photographs of liver sections with TUNEL staining are shown. The scale bar indicates 50 µm. Right, the numbers of TUNEL-positive hepatocytes/HPF were counted in WT-livers (5 mice) and Spred2^−/−^-livers (6 mice). *P < 0.05 vs. WT-liver. (**b**) Activities of caspase-3, caspase-8 and caspase-9 in WT-livers and Spred2^−/−^-livers (10 mice, each group). *P < 0.05, ***P < 0.001 vs. WT-livers. (**c**) mRNA expressions of FasL in WT-livers and Spred2^−/−^-livers (10 mice, each group) were analysed by RT-qPCR. The expression levels of each mRNA were normalized to that of GAPDH. *P < 0.05 vs. WT-livers. (**d**) Liver extracts were immunoblotted using the indicated primary antibodies. Upper, representative immunoblot data from two independent analyses of lysates from different mice. Lower, band densities were digitised and semi-quantitated. Full-length blots/gels are presented in Supplementary Figure 2. *P < 0.05 vs. WT-livers.

To determine the contribution of Spred2 to D-GalN/LPS-hepatotoxicity, we examined Spred2^−/−^ mice. Spred2 was constitutively expressed in wild-type (WT)-livers, but not in Spred2^−/−^-livers, as assessed by quantitatived RT-PCR (not shown). As shown in Fig. 1a, alanine aminotransferase (ALT) level, a specific marker of liver injury, was significantly increased in Spred2^−/−^ mice compared to in WT mice. Histologically, centrilobular necrosis with haemorrhage was much severer in Spred2^−/−^-livers than in WT-livers (Fig. 1b). Flow cytometry analyses revealed that the numbers of total leukocytes (CD45^+^), neutrophils (CD45^+^Ly6G^+^CD11b^+^), and macrophages (CD45^+^F4/80^+^CD11b^+^) were significantly higher in Spred2^−/−^ mice than in WT mice (2.0-fold increase in neutrophils and macrophages) (Fig. 1c). There were no significant differences in the basal numbers of leukocyte populations between groups (data not shown). Terminal deoxynucleotidyl transferase dUTP nick end labeling (TUNEL) staining of liver sections at 5 h post D-GalN/LPS showed that apoptotic hepatocytes were increased in Spred2^−/−^-livers compared to in WT-livers (Fig. 2a). Caspases are a class of cysteine proteases involved in apoptosis. The activities of caspase-3 and caspase-8, but not caspase-9, were significantly augmented in Spred2^−/−^-livers relative to in WT-livers (Fig. 2b). FasL expressions at 5 h post D-GalN/LPS were increased (Fig. 2c) whereas Bcl-2 levels were decreased (Fig. 2d) in Spred2^−/−^-livers relative to in WT-livers. Thus, Spred2^−/−^ mice developed exacerbated liver injury represented by augmented hepatocyte damage and hepatic inflammation after D-GalN/LPS challenge.

### ERK-MAPK pathway is augmented in Spred2^−/−^ mice

**Figure 3:**
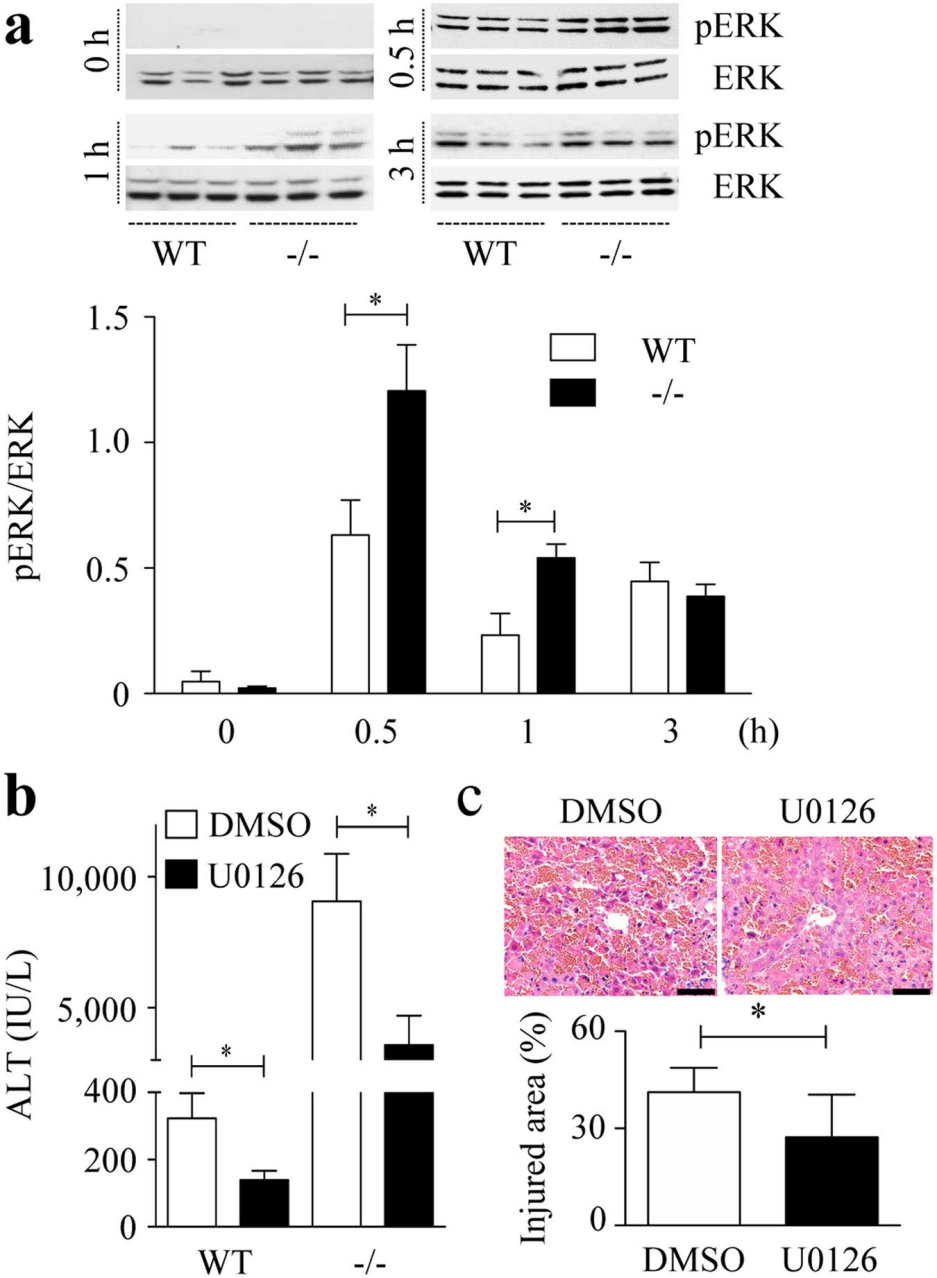
ERK-MAPK pathway is augmented in Spred2^−/−^ mice. (**a**) WT and Spred2^−/−^ mice were i.p. injected with D-GalN (400 mg/kg)/LPS (20 µg/kg), and sacrificed at indicated time intervals after injection. Upper, representative immunoblot data from two independent analyses of lysates from different mice. Lower, band densities were digitised and semi-quantitated (3–6 mice, each group). Full-length blots/gels are presented in Supplementary Figure 3. *P < 0.05 vs. WT-livers. (**b**,**c**) Mice were i.p. treated with 0.5 μM U0126 (7 mice) or vehicle control (DMSO, 8 mice), 2 h before D-GalN/LPS administration. At 5 h after D-GalN/LPS challenge, the mice were sacrificed. (**b**) Serum levels of ALT were measured. *P < 0.05 vs. WT mice. (**c**) Upper, representative photographs of the liver HE sections from Spred2^−/−^ mice are shown. The scale bar indicates 50 µm. Lower, injured area with haemorrhage was semi-quantitated. *P < 0.05 vs. WT livers.

In accordance with a previous report^15^, ERK activation, indicated by phosphorylated-ERK, was observed in WT-livers after D-GalN/LPS challenge, which was significantly augmented in Spred2^−/−^-livers (Fig. 3a). We next administered U0126, an inhibitor of the ERK-MAPK pathway that blocks the kinase activity of MAP kinase kinase (MAPKK or MEK 1/2) 2 h prior to D-GalN/LPS challenge. At 5 h after D-GalN/LPS challenge, mice were euthanized and the serum levels of ALT were examined. As shown in Fig. 3b, U0126 treatment reduced ALT levels to 42.9% in WT mice and 39.4% in Spred2^−/−^ mice (Fig. 3b). Histologically, centrilobular necrosis with haemorrhage was improved by U0126 treatment in Spred2^−/−^-livers (Fig., 3c). These results indicate that hepatotoxicity was largely dependent on the ERK-MAPK pathway, and the augmented liver injury in Spred2^−/−^ mice was due to upregulated ERK activation.

### Enhanced TNFα is responsible for increased ALI in Spred-2^−/−^ mice

**Figure 4:**
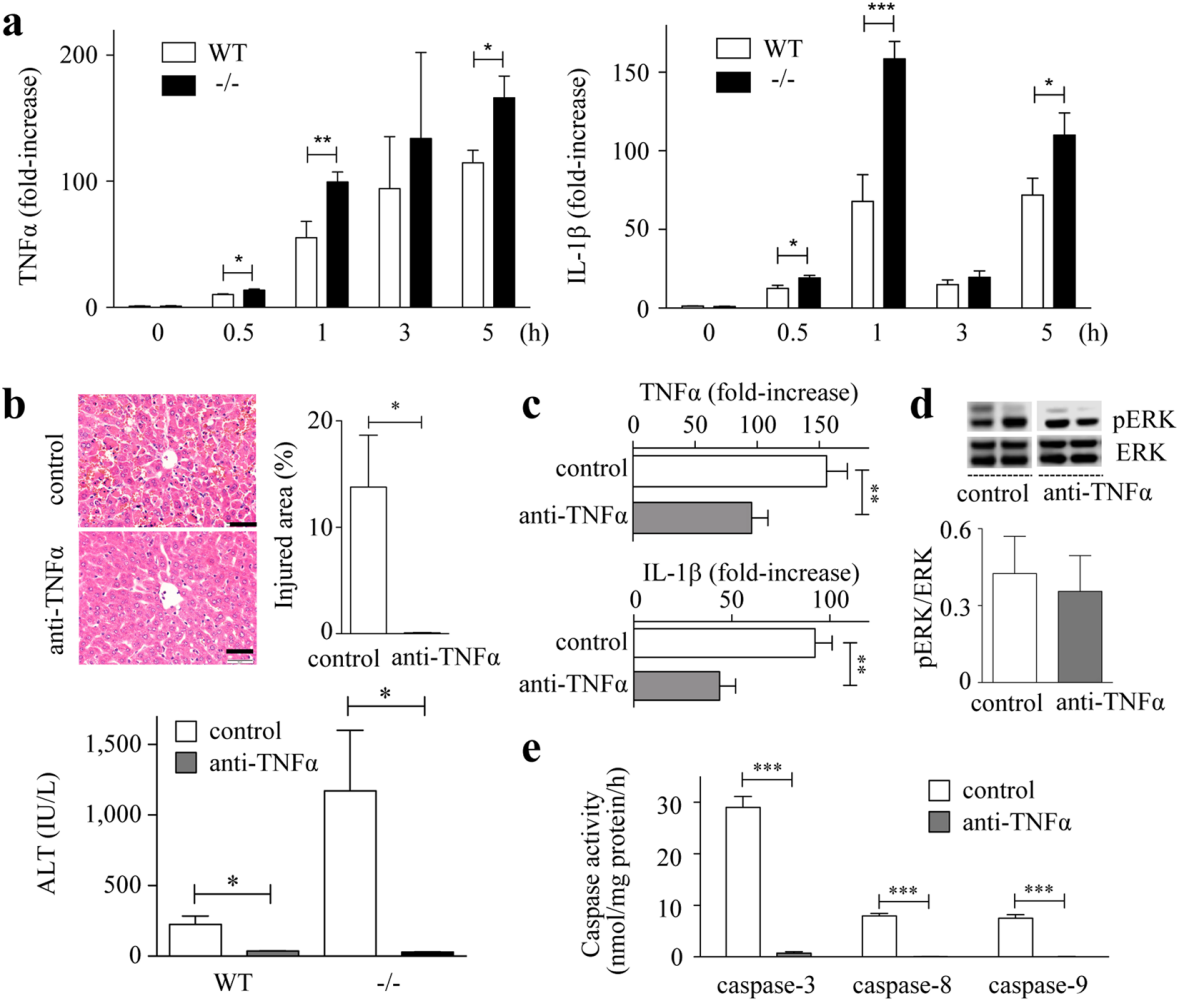
Enhanced TNFα production is responsible for increased acute liver injury in Spred2^−/−^ mice. (**a**) WT and Spred2^−/−^ mice were i.p. injected with D-GalN (400 mg/kg)/LPS (20 µg/kg), and the mice were sacrificed at indicated time intervals after injection. mRNA expression of TNFα (left) and IL-1β (right) in WT-livers and Spred2^−/−^-livers (3–9 mice, each group) was analysed by RT-qPCR. Expression levels of each mRNA were normalized to that of GAPDH. *P < 0.05, **P < 0.01, ***P < 0.001 vs. WT-livers. (**b**,**c**,**d**,**e**) Neutralizing anti-murine TNFα IgG or control IgG (each 750 µg/mouse) was i.p. administered into WT and Spred2^−/−^ mice 14 h before D-GalN/LPS administration. At 5 h after D-GalN/LPS challenge, the mice were sacrificed. (**b**) Upper left, representative photographs of liver HE sections from Spred2^−/−^ mice were shown. The scale bar indicates 50 µm. Upper right, injured area with haemorrhage from Spred2^−/−^ mice was semi-quantitated (4 mice, each). *P < 0.05 vs. WT livers. Lower, serum levels of ALT in WT mice (6 each) and Spred2^−/−^ mice (4 each) were measured. *P < 0.05 vs. control. (**c**) mRNA expression of endogenous TNFα (upper) and IL-1β (lower) in Spred2^−/−^-livers (4 mice, each group) was analysed by RT-qPCR. Expression levels of each mRNA were normalized to that of GAPDH. **P < 0.01 vs. control. (**d**) Upper, representative immunoblot data from different mice. Lower, band densities were digitised and semi-quantitated (4 mice, each group). Full-length blots/gels are presented in Supplementary Figure 4. (**e**) Activities of caspase-3, caspase-8 and caspase-9 in Spred2^−/−^-livers were measured (4 mice, each group). ***P < 0.001 vs. control.

**Figure 5:**
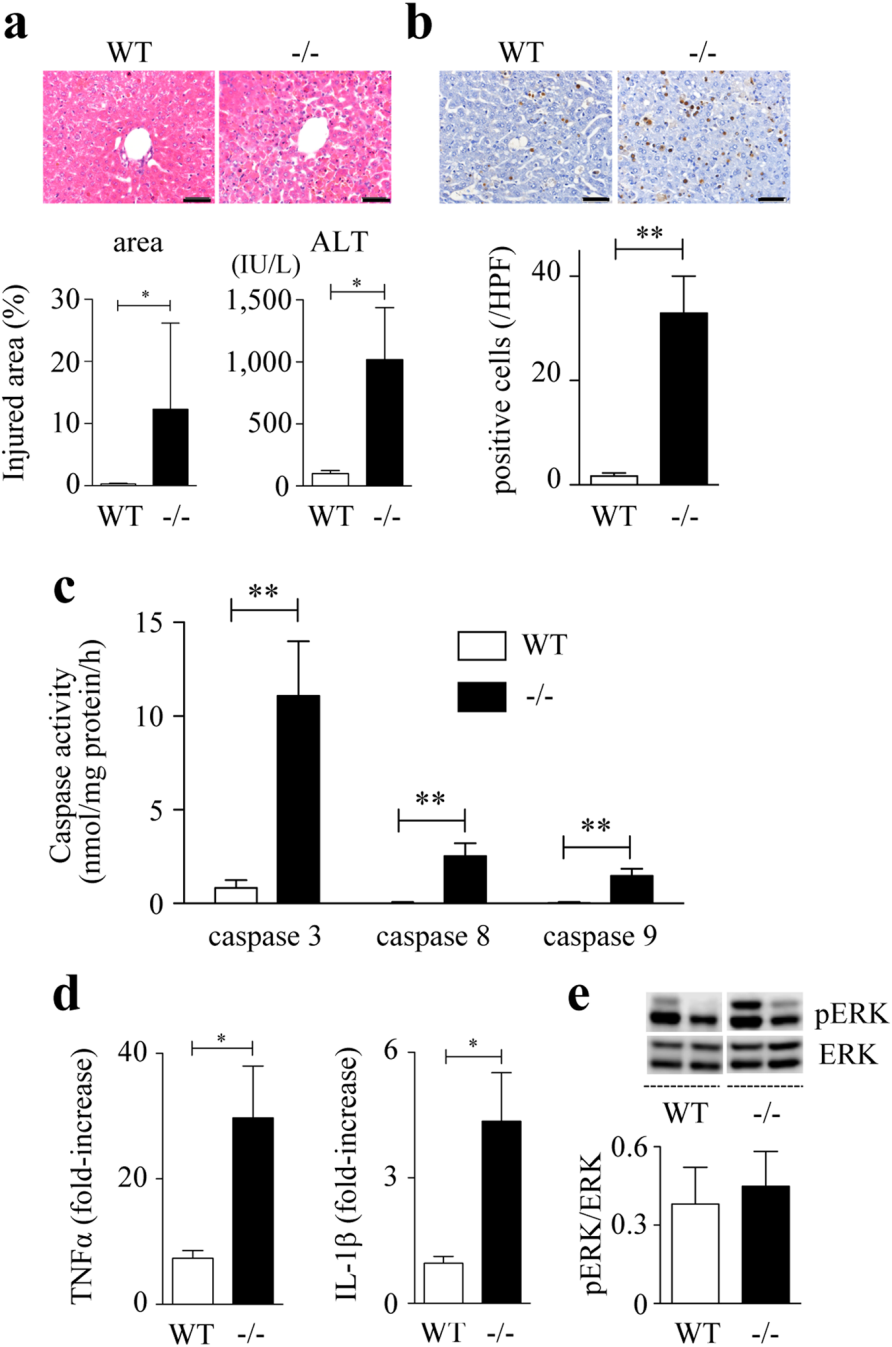
D-GalN/TNFα-induced liver injury. WT and Spred2^−/−^ mice were injected with D-GalN (700 mg/kg, i.p.)/TNFα (10 µg/kg, i.v.), and sacrificed at 6 h after challenge. (**a**) Upper, representative photographs of liver HE sections are shown. The scale bar indicates 50 µm. Lower left, injured area with haemorrhage was semi-quantitated (8 mice, each). Lower right, serum levels of ALT in WT mice (8 each) and Spred2^−/−^ mice (6 each) were measured. *P < 0.05 vs. WT mice. (**b**) Upper, representative photographs of liver sections with TUNEL staining are shown. The scale bar indicates 50 µm. Lower, the numbers of TUNEL-positive hepatocytes/HPF were counted in WT-livers and Spred2^−/−^-livers (6 mice, each group). **P < 0.01 vs. WT-liver. (**c**) Activities of caspase-3, caspase-8 and caspase-9 in WT-livers and Spred2^−/−^-livers were measured (8 mice, each group). **P < 0.01 vs. WT-liver. (**d**) mRNA expression of endogenous TNFα (left) and IL-1β (right) in WT-livers and Spred2^−/−^-livers (8 mice, each group) was analysed by RT-qPCR. Expression levels of each mRNA were normalized to that of GAPDH. *P < 0.05 vs. WT-liver. (**e**) Upper, representative immunoblot data from different mice. Lower, band densities were digitised and semi-quantitated (8 mice, each group). Full-length blots/gels are presented in Supplementary Figure 5.

The cytokine response, particularly those of the pro-inflammatory cytokines TNFα and IL-1β, is crucial in D-GalN/LPS-hepatotoxicity^8,20^. We therefore investigated the hepatic levels of TNFα and IL-1β after D-GalN/LPS challenge. The data in Fig. 4a demonstrate that the expressions of TNFα and IL-1β in the livers were increased after D-GalN/LPS challenge, both of which were significantly enhanced in Spred2^−/−^-livers. Because D-GalN/LPS-induced ALI largely depends on TNFα^8^, we neutralized TNFα using anti-TNFα antibody. The antibody was intraperitoneally (i.p.) injected 14 h prior to D-GalN/LPS challenge and the mice were sacrificed at 5 h after D-GalN/LPS challenge. Neutralization of TNFα dramatically ameliorated ALI, as demonstrated by the histology results and serum levels of ALT in both WT and Spred2^−/−^ mice (Fig. 4b). We also examined whether the production of TNFα might cause the endogenous expression of TNFα and IL-1β that could also induce liver injury. As shown in Fig. 4c, neutralization of TNFα reduced the expression of TNFα and IL-1β mRNA at 5 h after D-GalN/LPS challenge by 40% and 53%, respectively. Thus, the expression of TNFα and IL-1β appeared to be partly regulated by the production of endogenous TNFα in response to D-GalN/LPS. Under the experimental conditions, no changes were observed in ERK activation (Fig. 4d) in Spred2^−/−^-livers, suggesting that TNFα produced in an early stage stimulates subsequent endogenous TNFα and IL-1β without relation to ERK activation. In addition, the activities of caspases were almost completely reduced by anti-TNFα antibody in Spred2^−/−^-livers (Fig. 4e). When mice were challenged with D-GalN/TNFα, compared to WT mice, Spred2^−/−^ mice showed drastically enhanced ALI, as shown by histology and ALT levels in the serum (Fig. 5a). The numbers of apoptotic cells in Spred2^−/−^-livers were significantly higher than those in WT-livers (Fig. 5b). Furthermore, Spred2^−/−^-livers showed increased activities of caspases compared to those in WT-livers (Fig. 5c). D-GalN/TNFα challenge induced endogenous expressions of TNFα and IL-1β in WT-livers, which were significantly increased in Spred2^−/−^-livers (Fig. 5d). Under the conditions, there were no differences in the ERK activation between the groups (Fig. 5e), suggesting that augmented endogenous cytokine expressions by D-GalN/TNFα were independently regulated by ERK-MAPK pathway. The TNFα response during D-GalN/LPS-hepatotoxicity is regulated by its receptor expression by hepatocytes; however, TNF receptor 1 expression during ALI was similar between WT- and Spred2^−/−^-livers (Supplemental Fig. 1). Altogether, these results indicate that augmented expressions of endogenous TNFα in Spred2^−/−^-livers were responsible for the enhanced ALI after D-GalN/LPS challenge.

### Kupffer cells play a causative role in D-GalN/LPS-induced ALI

**Figure 6:**
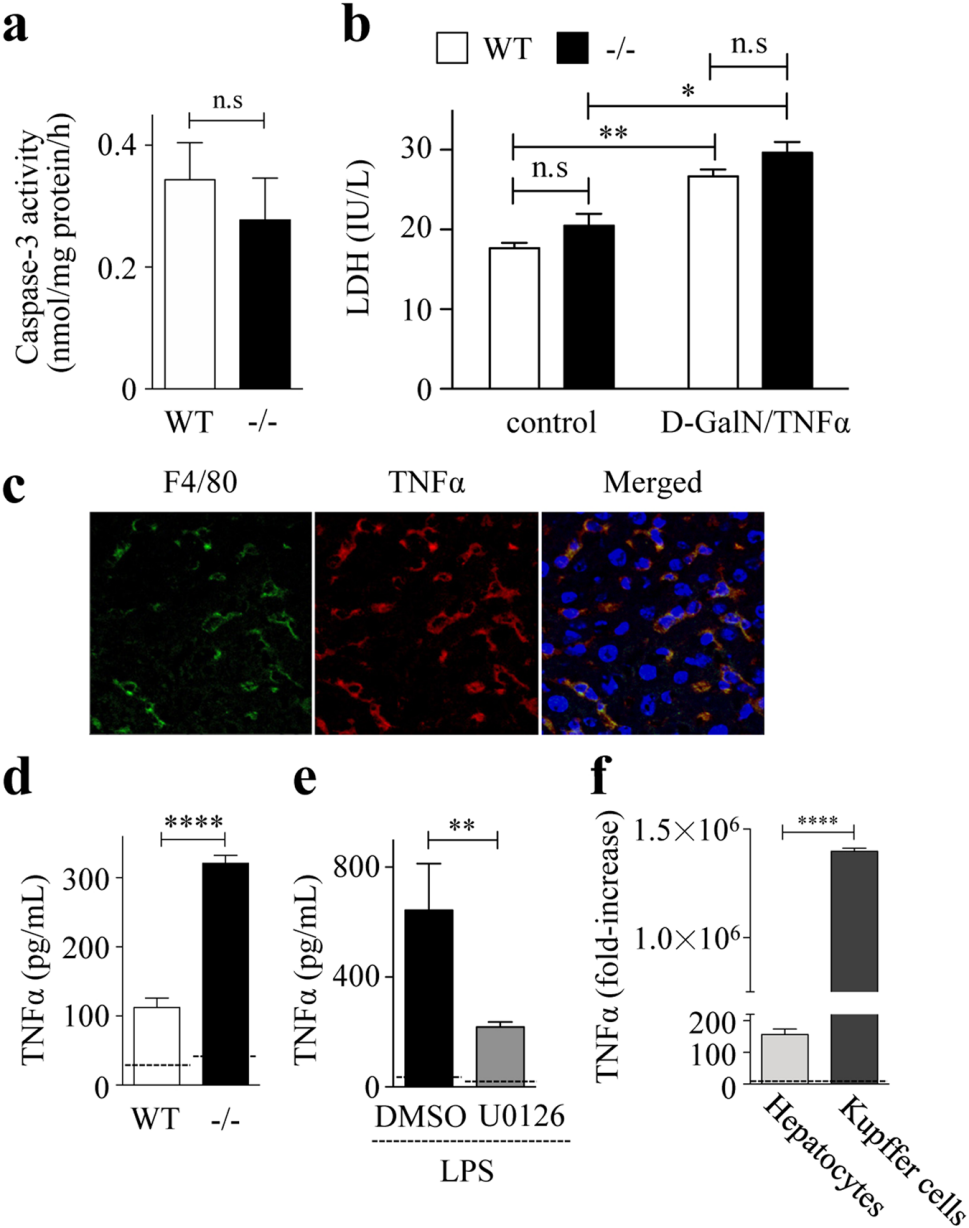
Kupffer cells play a causative role in D-GalN/LPS-induced ALI. (**a**,**b**) Hepatocytes were isolated from WT and Spred-2^−/−^ mice (3 mice each group), and the cells were stimulated with D-galactosamine (1 mg/mL) and TNFα (10 ng/mL) for 48 h. (**a**) Caspase-3 activity in WT-livers and Spred2^−/−^-livers was measured. n.s; not significant. (**b**) The culture supernatants were harvested and levels of lactate dehydrogenase (LDH) were measured. n.s; not significant. *P < 0.01, **P < 0.01 vs. control. (**c**) Spred2^−/−^ mice were i.p. injected with D-GalN (400 mg/kg)/LPS (20 µg/kg) and sacrificed at 2 h after challenge. Shown are representative frozen liver sections stained for F4/80 (green), TNFα (red), and DAPI (blue). A merged image is shown at the right end. (**d**) Kupffer cells were isolated from non-treated WT and Spred2^−/−^mice (3 mice, each group) and stimulated with or without LPS (100 ng/mL) for 6 h at 37 °C. TNFα level in the culture supernatants was measured by ELISA. Dotted line indicates medium control. ****P < 0.0001 vs. WT mice. (**e**) Spred2^−/−^-Kupffer cells were pre-treated with U0126 (10 μM) or DMSO 1 h prior to LPS (100 ng/mL) stimulation. At 6 h after LPS stimulation, culture supernatants were harvested and the levels of TNFα were measured by ELISA (4 samples, each group). Dotted line indicates medium control. **P < 0.01 vs control. (**f**) Hepatocytes and Kupffer cells were isolated from Spred2^−/−^ mice and the cells were stimulated with LPS (100 ng/mL) for 6 h at 37 °C. mRNA expressions of TNFα were analysed by RT-qPCR. Expression levels of each mRNA were normalized to that of GAPDH. Dotted line indicates expression level from non-stimulated hepatocytes. ****P < 0.0001 vs. hepatocytes.

Considering that Spred2 is also expressed by hepatocytes, Spred2 deficiency in hepatocytes could directly affect liver injury. To investigate this hypothesis, isolated hepatocytes from WT and Spred2^−/−^ mice were stimulated *in vitro*. Upon stimulation with D-GalN/TNFα, WT- and Spred2^−/−^-hepatocytes showed equivalent damage, as evidenced by caspase-3 activity (Fig. 6a) and lactate dehydrogenase release from hepatocytes (Fig. 6b). We next investigated the cellular source of TNFα in the livers. Immunofluorescence double staining demonstrated that F4/80^+^ cells, likely Kupffer cells, were positive for TNFα after D-GalN/LPS challenge (Fig. 6c). Among F4/80^+^ Kupffer cells, CD11b^+^ Kupffer cells are known to produce TNFα^21^. We next isolated CD11b^+^ Kupffer cells from non-treated WT and Spred2^−/−^ mice and the cells were stimulated with LPS *in vitro*. TNFα production was significantly higher in Spred2^−/-^Kupffer cells than in WT controls (Fig. 6d). The augmented production of TNFα in Spred2^−/−^-Kupffer cells was decreased by U0126 (Fig. 6e). Thus, Spred2^−/−^-Kupffer cells produce higher levels of TNFα upon stimulation with LPS, possibly through an enhanced activation of ERK-MAPK pathway. It is possible that hepatocytes could also produce TNFα, leading to hepatocyte damage. To examine this, TNFα mRNA expressions were compared after LPS-stimulation between hepatocytes and Kupffer cells from Spred2^−/−^ mice. The data in Fig. 6f showed that TNFα expression in Kupffer cells was 9 × 10^3^-flod higher than in hepatocytes. Furthermore, TNFα protein in the culture supernatants from Spred2^−/−^-hepatocytes were below detection level (by ELISA, not shown) although appreciable level of TNFα was produced from Kupffer cells (Fig. 6d). These results suggest that Kupffer cells were major cell source for TNFα.

## Discussion

Despite advances in medicine and health care, the management of ALI is currently a significant challenge. D-GalN/LPS-induced ALI in mice is a clinically relevant and widely used model of ALI characterized by apoptotic cell death and severe inflammation^22^. Because robust ERK activation was observed in D-GalN/LPS-induced ALI^15^, we hypothesized that Spred2, a negative regulator of ERK/MAPK, is involved in the hepatotoxicity. We demonstrated that Spred2 deficiency exacerbated the hepatotoxicity. The aberrant hepatotoxicity was associated with enhanced ERK activation in the livers, and the MEK/ERK inhibitor U0126 diminished the augmented hepatotoxicity in Spred2^−/−^ mice. These data clearly demonstrate that over-action of ERK-MAPK is deleterious during ALI and endogenous Spred2 down-regulates the ERK/MAPK pathway.

D-GalN/LPS-induced ALI depends on endogenous TNFα^23^. TNFα-induced hepatocyte apoptosis is an early and causal event in hepatotoxicity^8^. When hepatocytes were stimulated with D-GalN/TNFα, caspase-3 activity in hepatocytes and lactate dehydrogenase release from hepatocytes were similar between WT and Spred2^−/−^ hepatocytes, indicating that hepatocyte damage caused by TNFα was comparable. We demonstrated that TNFα expression was significantly augmented in Spred2^−/−^-livers. Neutralization of TNFα nearly completely abrogated the enhanced hepatotoxicity in Spred2^−/−^ mice. Furthermore, TNFα at doses as low as 10 µg/kg (approximately 200 ng/mice) reproduced hepatotoxicity in Spred2^−/−^ mice in the presence of D-GalN. We also demonstrated that the production of TNFα caused the endogenous expression of TNFα and IL-1β that could induce liver injury. Thus, augmented TNFα expression in Spred2^−/−^-livers may be ascribed to exacerbated ALI in Spred2^−/−^ mice. Another possibility is that hypercoagulability caused by augmented TNFα contributes to hepatotoxicity development^24^. The ERK-MAPK pathway is involved in fibrin accumulation in LPS-induced ALI caused by ethanol, which is completely abrogated in TNF receptor-1^−/−^ mice^25^. TNFα is a potent activator of neutrophils, which may cause hepatocyte damage^26^. Further studies are necessary to identify the precise molecular mechanisms involved.

Notably, TNFα expression at 1 h post-D-GalN/LPS injection in Spred-2^−/−^-livers was 1.8-fold higher than in WT-livers, whereas a 2.3-fold increase was observed for the expression of IL-1β. Because IL-1β is important in the pathogenesis of D-GalN/LPS-induced ALI^27^, the augmented IL-1β appeared to contribute to enhanced hepatotoxicity in Spred2^−/−^ mice. Interestingly, IL-1β expression was decreased by 53% by neutralization of TNFα in D-GalN/LPS-induced ALI, and D-GalN/TNFα increased endogenous expression of IL-1β and TNFα. These results suggest that IL-1β expression was mostly dependent on TNFα.

Kupffer cells reside within the liver sinusoid and serve as gatekeepers. Kupffer cells can be activated by various stimuli including LPS, resulting in the release of an array of inflammatory mediators^28,29^. Here, we demonstrated that Kupffer cells produced TNFα in D-GalN/LPS-induced hepatotoxicity. We showed that Spred2^−/−^-Kupffer cells produced more TNFα compared to WT-Kupffer cells after LPS stimulation. Sperd2^−/−^–hepatocytes may also contribute to the augmented TNFα in the Spred2^−/−^-livers; however, this is unlikely because TNFα expression in hepatocytes was extremely lower than in Kupffer cells. We showed that the MEK inhibitor U0126 decreased the level of TNFα in Spred2^−/−^-Kupffer cells. ERK activation is known to be involved in LPS-stimulated TNFα production by Kupffer cells^30^. Thus, augmented TNFα production in Spred-2^−/−^-livers appeared to result from enhanced ERK activation in Kupffer cells. Similarly, we recently demonstrated that alveolar macrophages and bone marrow-derived macrophages from Spred2^−/−^ mice produced significantly higher levels of TNFα relative to control mice upon stimulation with LPS. Spred2 knockdown promoted ERK activation and increased TNFα levels in RAW264.7 cells^19^.

There were several limitations to this study. First, LPS activates not only ERK, but also JNK and p38 in Kupffer cells^30^. Liver injury and mortality caused by D-GalN/LPS was markedly decreased in JNK2^−/−^ mice. Because U0126 inhibits MEK1 and MEK2 with negligible effects on other protein kinases, such as ERK, p38, and JNK^31^, an inhibitor other than U0126 and/or a direct inhibitor against each MAPK should be used to elucidate the precise mechanisms involved. Second, cells expressing Spred2 in the livers have not been characterized because there are no effective antibodies against murine Spred2 available for use in immunohistochemistry. Third, in addition to MAPK, the LPS/TLR4- and TNFα/TNF receptor 1 signalling pathways involve NF-κB-signaling^9,32^. Considering the complexity of intracellular signalling, cross-talk between ERK-MAPK and NF-κB must be elucidated. Finally, we demonstrated that Spred-2 deficiency enhanced ALI, indicating that D-GalN/LPS-induced ALI can be treated by Spred-2 supplementation *in vivo*. Further studies are necessary to address these issues.

In conclusion, we showed that Spred2 deficiency exacerbates hepatotoxicity via increased production of TNFα in a murine model of D-GalN/LPS-induced ALI. D-GalN/LPS-induced ALI showed severe hepatic damage accompanied by apoptotic and necrotic changes in the liver, which is similar to that in human liver failure^8,33^. A better understanding of the signalling pathway involved in this animal model may provide insight and lead to identification of potential therapeutic targets.

## Materials and Methods

### Reagent

LPS (*Escherichia coli* 0111:B4) and D-GalN were purchased from Sigma-Aldrich (St. Louis, MO, USA). U0126 was from Promega (Madison, WI, USA). Antibodies to p44/42 MAPK (ERK1/2), phospho-p44/42 MAPK (ERK1/2), Bcl-2, and GAPDH were purchased from Cell Signaling Technology (Danvers, MA, USA). The antibody to TNF receptor 1 was from Santa Cruz Biotechnology (Santa Cruz, CA, USA). For flow cytometry analysis, anti-mouse CD16/32, FITC anti-mouse Ly6G (1A8), PerCP/Cy5.5 anti-mouse CD11b (M1/70)(BioLegend, San Diego, CA, USA), PE anti-mouse F4/80 (BM3) (eBioscience, San Diego, CA, USA), PE-Cy7 anti-mouse CD45 (30-F11)(BD Pharmingen, San Jose, CA, USA), and PE-CY5.5-conjugated PI (Trevigen, Gaithersburg, MD, USA) were employed. Murine recombinant TNFα was purchased from R&D Systems (Minneapolis, MN, USA).

### Mice

Spred2^−/−^ mice backcrossed onto a C57BL/6 background were kindly provided by Dr. Akihiko Yoshimura (Keio University, Japan)^34,35^. No Spred2 expression was detected in circulating leukocytes, lungs, livers and kidneys from Spred2^−/−^ mice, as assessed using TaqMan RT-qPCR (data not shown). C57BL/6 J mice were used as WT mice. These mice were bred and maintained under continuous 12-h light and 12-h dark cycle under specific pathogen-free conditions at the Department of Animal Resources, Okayama University (Okayama, Japan). Female mice (7–10 weeks) were used in this experiment and given a standard diet and water *ad libitum*. The animal care and use committee at Okayama University approved all experiments in this study. All experiments were performed in accordance with relevant guidelines and regulations.

### Acute liver injury model

Mice were given intraperitoneal (i.p.) injection of LPS (20 µg/kg) and D-GalN (400 mg/kg) dissolved in phosphate-buffered saline (PBS). In some experiments, mice were pre-treated by i.p. injection of D-GalN (700 mg/kg) 30 min before intravenous (i.v.) injection of mouse recombinant TNFα (10 µg/kg). In a different set of experiments, mice were treated i.p. with 0.5 μM U0126 or vehicle control (DMSO) 2 h before D-GalN/LPS administration. To neutralize endogenous TNFα, neutralising rabbit anti-murine TNFα IgG (750 µg/mouse) was administered i.p. 14 h before D-GalN/LPS administration. A high titered rabbit anti-murine TNFα was prepared as described^36^ and IgG was purified using Protein A IgG purification kit (Thermo fisher scientific, Oregon, USA). Control rabbit IgG was used as a control. At different time intervals, mice were anesthetized, bled, and sacrificed. Livers were perfused with sterile saline, and excised. A part of the livers was snap-frozen in liquid nitrogen and stored at −80 °C for subsequent analyses. Another part was fixed in 10% formalin and embedded in paraffin, and the liver sections were histologically examined. Injured area with haemorrhage in captured images were semi-quantitated using NIH’s ImageJ, a free image analysis software. A whole liver was used for leukocyte differential cell analysis in the liver by flow cytometry. Serum levels of ALT were measured using standardized techniques.

### Detection of apoptosis and caspase activity

Apoptotic cells in the liver were detected by TUNEL assay using an *in situ* apoptosis detection kit (TACS2 TdT-DAB; Trevigen) as per the manufacturer’s instructions and the positive cells were counted under a microscope. To measure caspase-3, caspase-8, and caspase-9 activities, a colorimetric assay kit (MBL, Nagoya, Japan) for each caspase was used. Livers were homogenized in the lysis buffer provided in the kit and the activities in cytosolic extracts (150 µg protein) were measured. Protein concentrations in the extracts were measured in a protein-dye binding assay (Bio-Rad Laboratories, Hercules, CA, USA).

### Isolation of hepatic leukocytes and Kupffer cells

Livers were perfused with 50 mL saline solution, minced, suspended in 5 mL Hank’s Balanced Salt Solution (HBSS) (20 µg/mL collagenase, 150 U/mL DNase I, and 20 mM HEPES, pH = 7.4), and incubated for 20 min at 37 °C in a gentleMACS™ Dissociator (Miltenyi Biotec GmbH, Bergisch Gladbach, Germany). Cell suspension was filtered through a 40-µm nylon mesh and centrifuged at 300 × *g* for 6 min. Cell pellets were suspended in Percoll (final concentration, 35% for leukocyte isolation; 33% for Kupffer cell isolation), and centrifuged at 370 × *g* and 500 × *g*, respectively, for 10 min. Cell pellets were washed once with PBS, resuspended in PBS and the cell number was counted with haemocytometer. More than 90% of the resulting cells were alive according to trypan blue exclusion. For Kupffer cell isolation, CD11b^+^ cells were isolated from the cells using anti-CD11b microbeads over MS^+^ MiniMACS separation columns (Miltenyi Biotec) according to manufacturer’s instructions. Most purified cells were alive (>90%, trypan blue exclusion) and were >90% CD11b^+^ cells.

### Isolation and culture of primary hepatocytes

Hepatocytes were isolated using a modified two-step collagenase perfusion technique as previously described^37,38^. Freshly isolated hepatocytes (>95% alive, trypan blue exclusion) were suspended in hepatocyte maintenance medium (Thermo fisher scientific) and placed on collagen I-coated 12 well plates (2 × 10^5^ cells/well). After overnight incubation, hepatocytes were stimulated with murine recombinant TNFα (10 ng/mL) and D-GalN (1 mg/mL) for 48 h. The culture supernatant was harvested and lactate dehydrogenase concentration was measured using standardized techniques.

### Flow cytometry

Cells were suspended in PBS containing 2% foetal bovine serum and 0.1% sodium azide, and stained with the indicated antibodies after 10 min of pre-incubation with CD16/CD32 antibody (Fc block) and fixed overnight using 1% formalin. Expression of each antigen was detected using a MACSQuant Analyzer (Miltenyi Biotec), and data were analysed using FlowJo software (Tree Star Inc., Ashland, OR, USA). The number of cell populations in each liver was calculated by multiplying total cell number by the respective percentage indicated.

### Kupffer cell stimulation

Isolated Kupffer cells were suspended in Dulbecco’s Modified Eagle’s medium (DMEN) solution, containing 10% foetal bovine serum, 1% non-essential amino acids, sodium pyruvate (1 mM), and penicillin (100 U/mL)/streptomycin (100 µg/mL). Cells were plated onto 24-well plates at a density of 2.5 × 10^5^ cells per well, incubated for 1 h, and non-adherent cells were washed away with DMEN and adherent Kupffer cells were stimulated with LPS (100 ng/mL).

### Real-time quantitative PCR (RT-qPCR)

Total RNA was isolated from whole livers and cultured cells using Trizol Reagent (Gibco BRL, Grand Island, NY, USA) and High Pure RNA Isolation kit (Roche Applied Science, Basel, Switzerland), respectively. First-strand cDNA was constructed from total RNA using the oligo (dT)_12–18_ primer. Real-time quantitative PCR analysis was performed using StepOne with Taqman PCR master mix (Applied Biosystems, Foster City, CA, USA). The primers used in this study were: GAPDH(Mn99999915_-_g1), TNFα (Mn00443258_–_m1), IL-1β (Mn00434228_–_m1), FasL (Mm00438864.m1), and Spred2 (Mn01223872_-_g1) (Applied Biosystems). The quantification of the genes of interests was normalized to GAPDH and expressed as fold-increases relative to the negative control for each treatment at each time point as previously described^39^.

### Western blotting

Livers were extracted in a lysis buffer (Cell Signaling Technology), and cleared supernatants were stored at −80 °C until use. Protein concentration in the lysates was measured by protein-dye binding assay (Bio-Rad Laboratories, Inc). Equal amounts (15 μg) of samples were fractionated by sodium dodecyl sulphate-polyacrylamide gel electrophoresis (Invitrogen, Carlsbad, CA, USA) and transferred to a nitrocellulose membrane. After overnight incubation with the appropriate primary antibody, the membrane was incubated with horseradish peroxidase-conjugated rabbit IgG antibody (Santa Cruz Biotechnology) and the presence of each protein was visualized with enhanced chemiluminescence detection reagents (ImmunoStar LD; Wako, Osaka, Japan). Blots were photographed and analysed with Image Studio software.

### Immunofluorescence assessment

Livers were frozen in O.C.T compound and sectioned at 4 μm-thick. After fixation with ice-cold acetone for 3 min, the sections were incubated with rat anti-mouse F4/80 (eBioscience) and goat anti-mouse TNFα antibody (R&D Systems) overnight at 4 °C. Tissue sections were then washed with TBS-T and incubated for 30 min with Alexa Fluor 647 rabbit anti-goat IgG and Alexa Fluor 568 Goat anti-rat IgG (Invitrogen). DAPI (4′,6-diamidino-2-phenylindole) was used for nuclear staining. The tissue sections were analysed using an Olympus confocal microscope system (Olympus, Tokyo, Japan).

### Enzyme-linked immunosorbent assay (ELISA)

Murine TNFα was measured using a standard sandwich ELISA as previously described^40,41^. The primary antibody, detection antibody, and recombinant TNFα were purchased from R&D Systems. The ELISAs employed in this study did not cross-react with other murine cytokines available.

### Statistics

Statistical significance was evaluated by analysis of variance. All data were expressed as the mean ± SEM. Differences of *p* < 0.05 were considered significant. All statistical calculations were performed using GraphPad Prism 6 (GraphPad Software, San Diego, CA, USA).

## Electronic supplementary material

Supplementary Information
