# Supplementary material for: Spred2 Deficiency Exacerbates D-Galactosamine/Lipopolysaccharide -induced Acute Liver Injury in Mice via Increased Production of TNFα
Source: Sci Rep. 2018 Jan 9;8:188. doi: 10.1038/s41598-017-18380-0 (PMC5760641; doi:10.1038/s41598-017-18380-0)
Supplement: Supplementary file 1 — Supplementary Information [file 41598_2017_18380_MOESM1_ESM.pdf]

Spred2 Deficiency Exacerbates D-*Galactosamine*/Lipopolysaccharide-induced Acute Liver Injury in Mice via Increased Production of TNFα

Xu Yang, Masayoshi Fujisawa, Teizo Yoshimura, Toshiaki Ohara, Miwa Sato, Megumi Mino, Thar Htet San, Tong Gao, Steven L. Kunkel and Akihiro Matsukawa

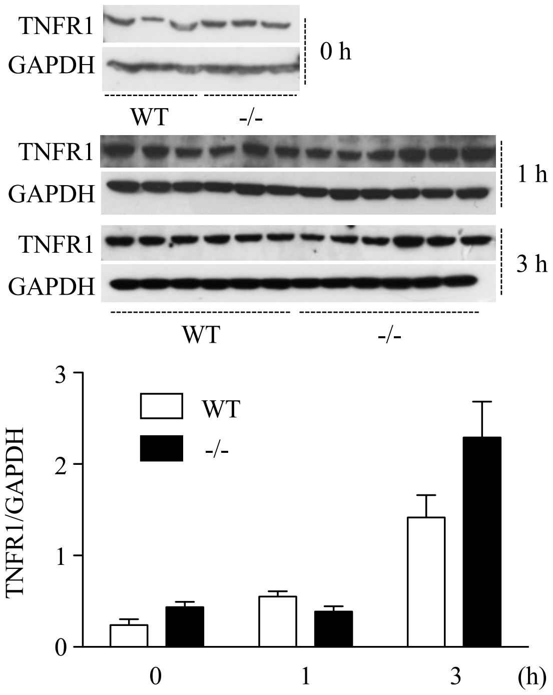

Supplemental Figure 1
WT and Spred-2$^{-/-}$ mice were intraperitoneally injected with D-galactosamine (400 mg/kg) and LPS (20 μg/kg). At appropriate time intervals, mice were sacrificed and the livers were resected. Livers were extracted and expressions of TNF receptor 1 (TNFR1) in liver extracts were analysed by western blotting. There were no differences between WT- and Spred-2$^{-/-}$-livers (time 0: 3 each, time 1 and 3 h: 6 mice each).

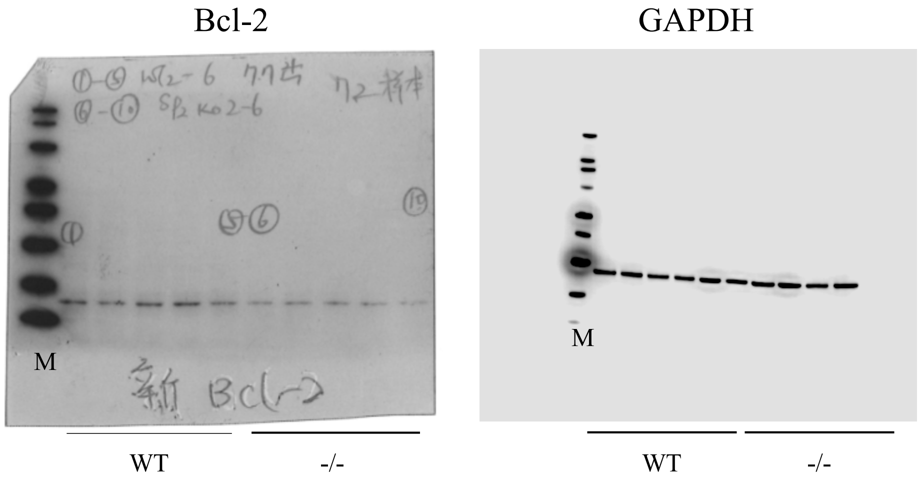

Supplemental Figure 2

Liver extracts at 5 h after injection of D-GalN/LPS were immunoblotted using the indicated primary antibodies. Uncropped images of the Figure 2d are shown. Left; Bcl-2, Right; GAPDH. M; Marker. Shown are 5 each from WT and Spred2$^{-/-}$-livers.

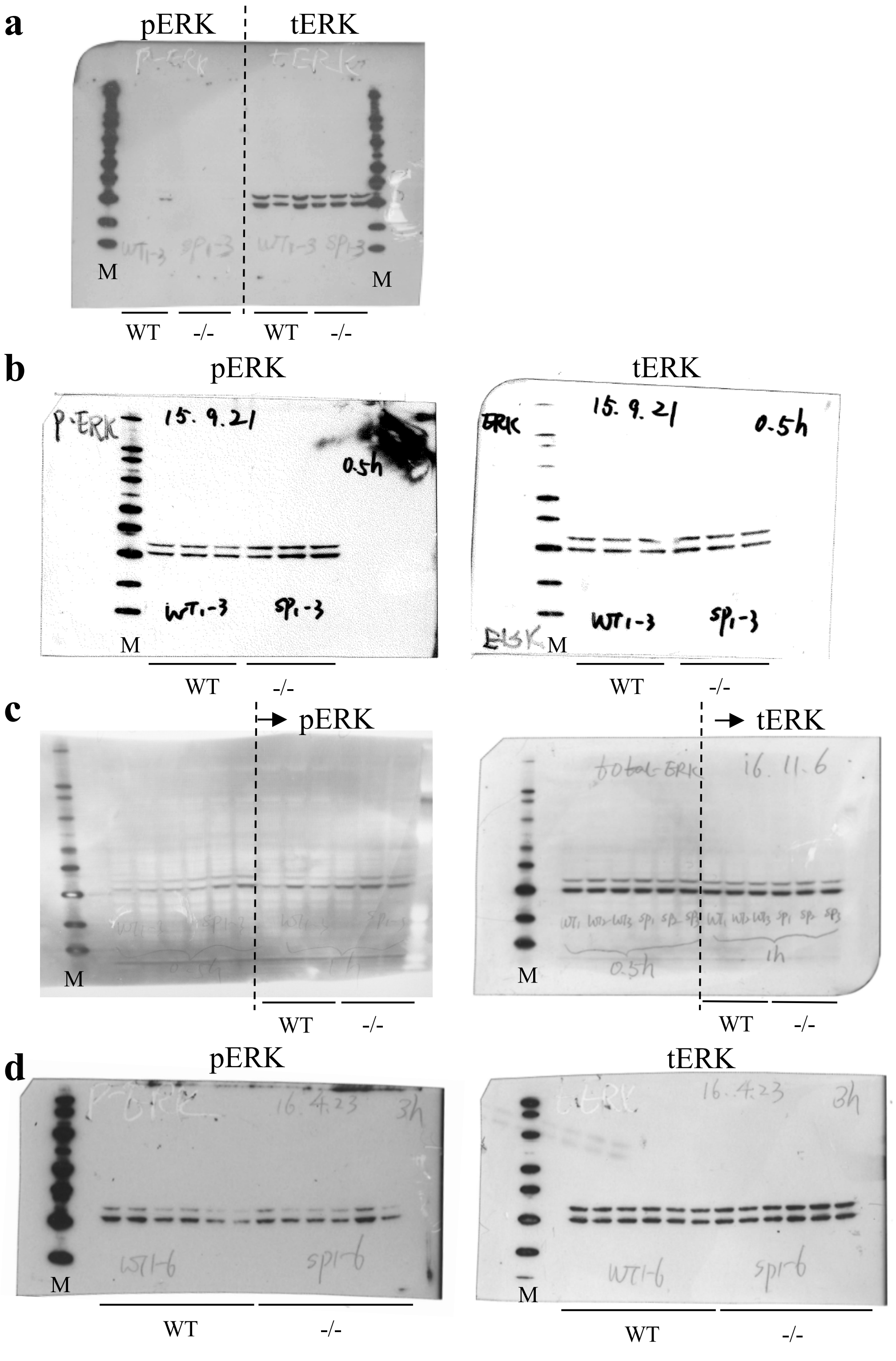

Supplemental Figure 3

Liver extracts at 0 h (a), 0.5 h (b), 1 h (c), and 3 h (d) after injection of D-GalN/LPS were immunoblotted using the indicated primary antibodies. Uncropped images of the Figure 3a are shown. M; Marker. Shown are 3 each (a-c) and 6 each (d) from WT and Spred2$^{-/-}$-livers.

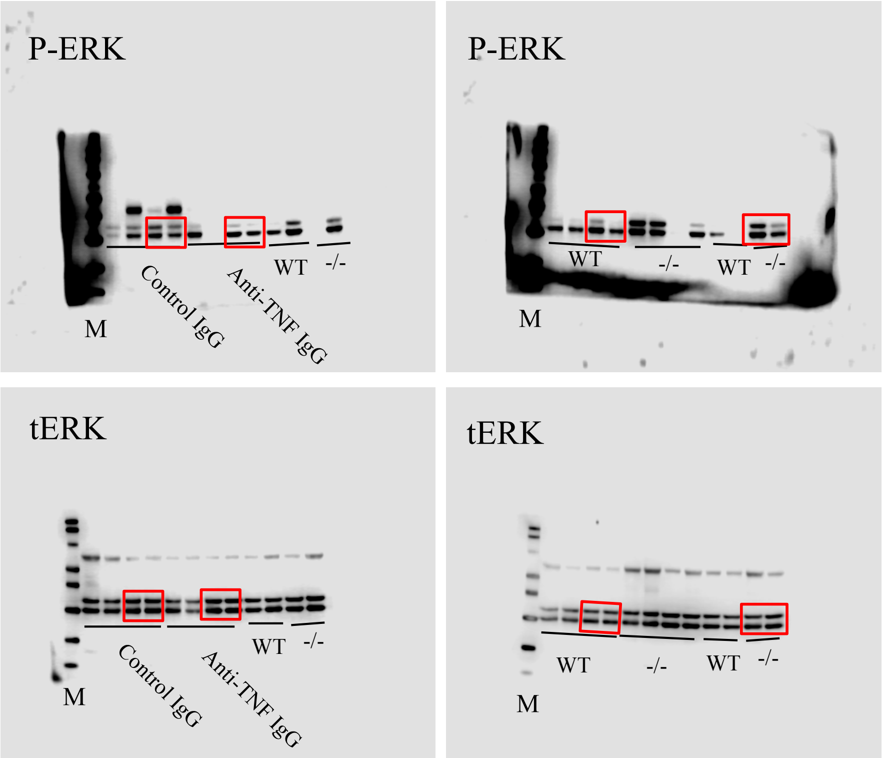

Supplemental Figure 4

Experiment 1: Neutralizing anti-murine TNFα IgG or control IgG (each 750 µg/mouse) was i.p. administered into Spred2$^{-/-}$ mice 14 h before D-GalN/LPS administration. At 5 h after D-GalN/LPS challenge, the mice were sacrificed. Liver extracts at 5 h after injection of D-GalN/LPS were immunoblotted using the indicated primary antibodies. Uncropped images (shown red square) of the Figure 4d are shown. M; Marker. Shown are 4 each from Spred2$^{-/-}$-livers.

Experiment 2: WT and Spred2$^{-/-}$ mice were injected with D-GalN (700 mg/kg, i.p.)/TNFα (10 µg/kg, i.v.), and sacrificed at 6 h after challenge. Liver extracts were immunoblotted using the indicated primary antibodies. Uncropped images (shown red square) of the Figure 5e are shown. M; Marker. Shown are 8 each from WT and Spred2$^{-/-}$-livers.

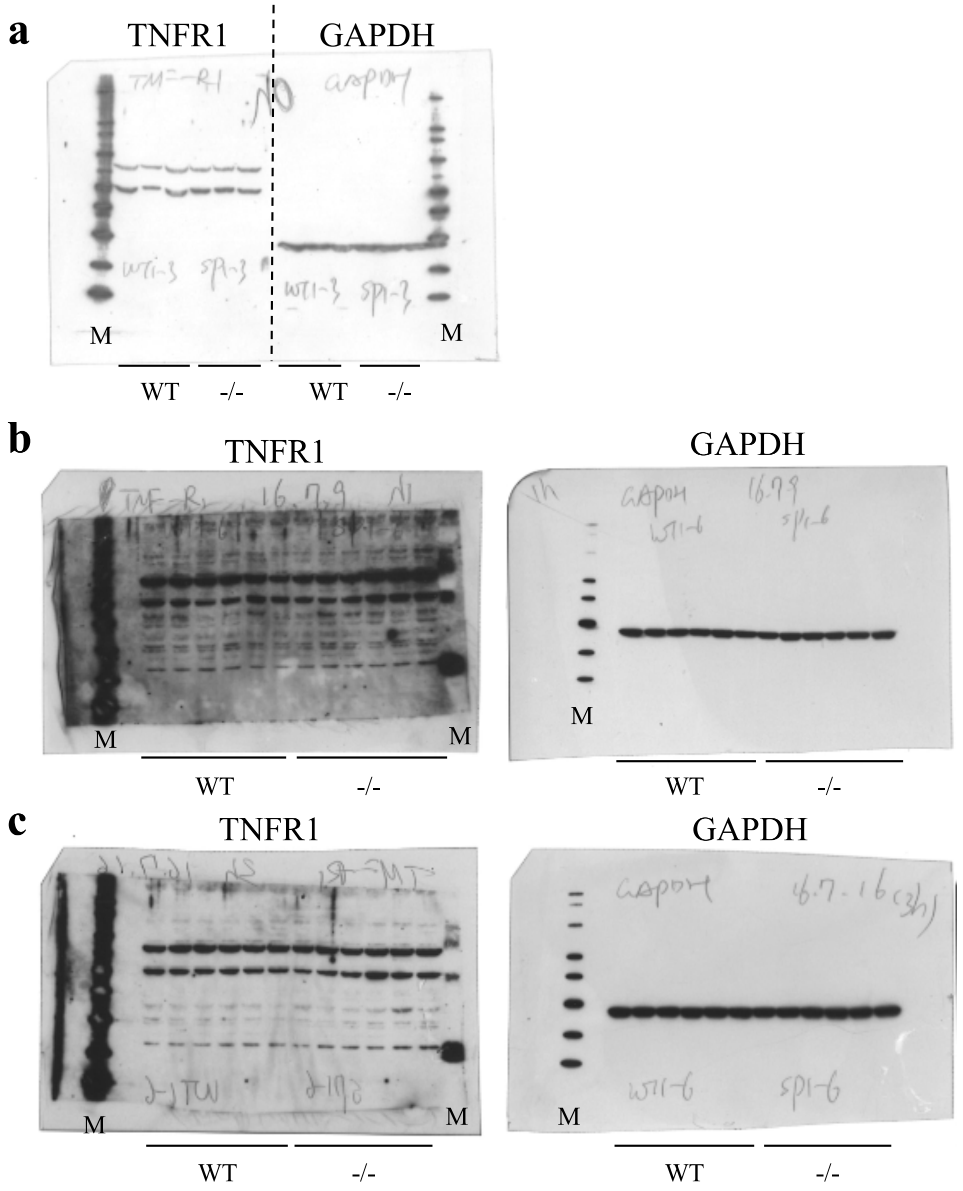

Supplemental Figure 5

Liver extracts at 0 h (a), 1 h (b), and 3 h (c) after injection of D-GalN/LPS were immunoblotted using the indicated primary antibodies. Uncropped images of the Supplemental Figure 1 are shown. M; Marker. Shown are 3 each from WT and Spred2$^{-/-}$-livers at 0 h (a), and 6 each at 1 (b) and 3 h (c).
